# Supplementary material for: Genetic suppression interactions are highly conserved across genetically diverse yeast isolates
Source: G3 (Bethesda). 2025 Mar 3;15(5):jkaf047. doi: 10.1093/g3journal/jkaf047 (PMC12060245; doi:10.1093/g3journal/jkaf047)
Supplement: jkaf047_Supplementary_Data [file jkaf047_supplementary_data.zip › Supplemental_Figures_G3-2025-405742.pdf]

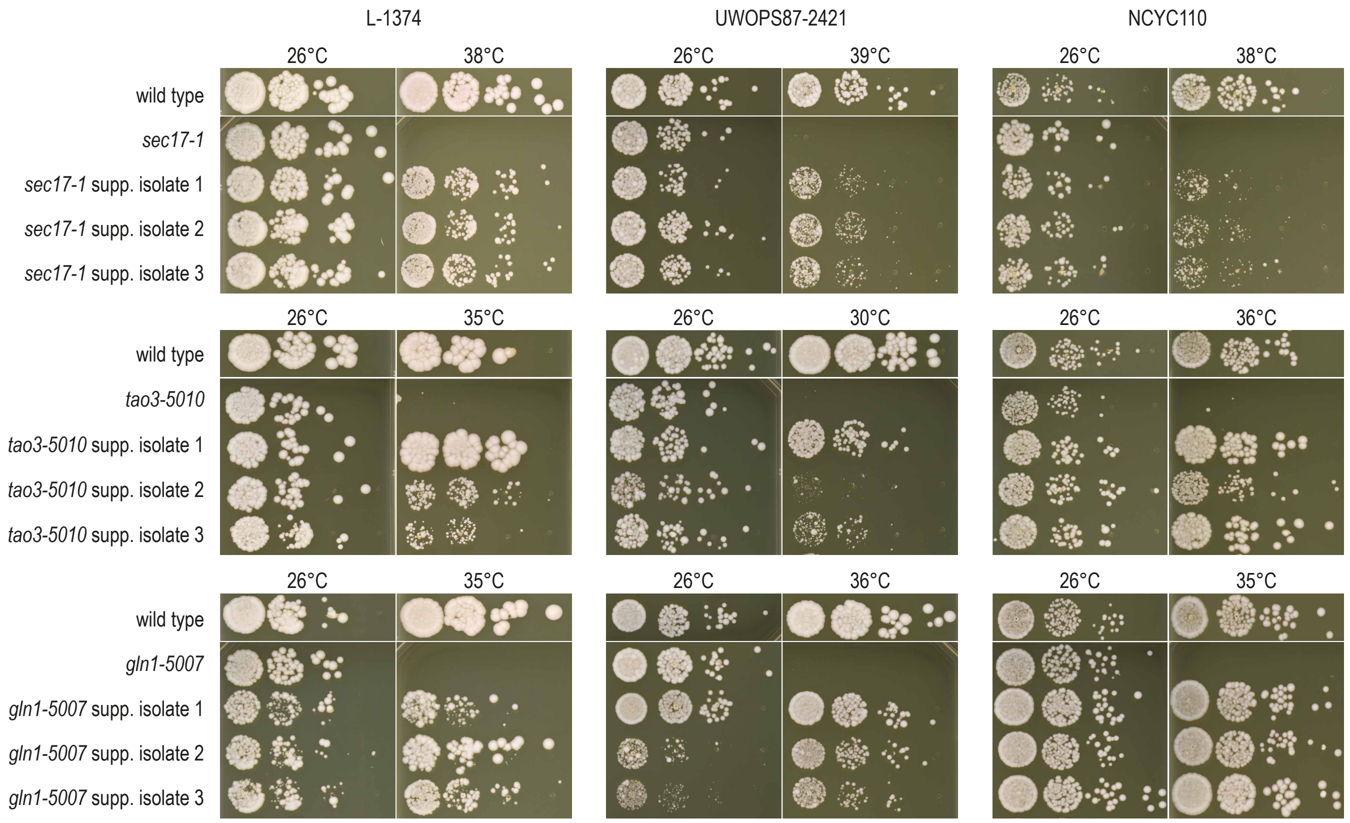

Fig. S1. Validating the suppression phenotype of isolated suppressor strains. Three TS alleles (*sec17-1*, *tao3-5010*, and *gln1-5007*) were introduced into three natural yeast isolates (L-1374, UWOPS87-2421, and NCYC110) and spontaneous suppressors of the TS phenotype were isolated. Cultures of the isolated suppressor strains, as well as of the corresponding parental TS strains without a suppressor, were grown until saturation, and a series of ten-fold dilutions was spotted on YPD plates. Plates were incubated at the indicated temperatures for two days. The wild-type natural isolates (without TS allele) were included on each plate as a control.

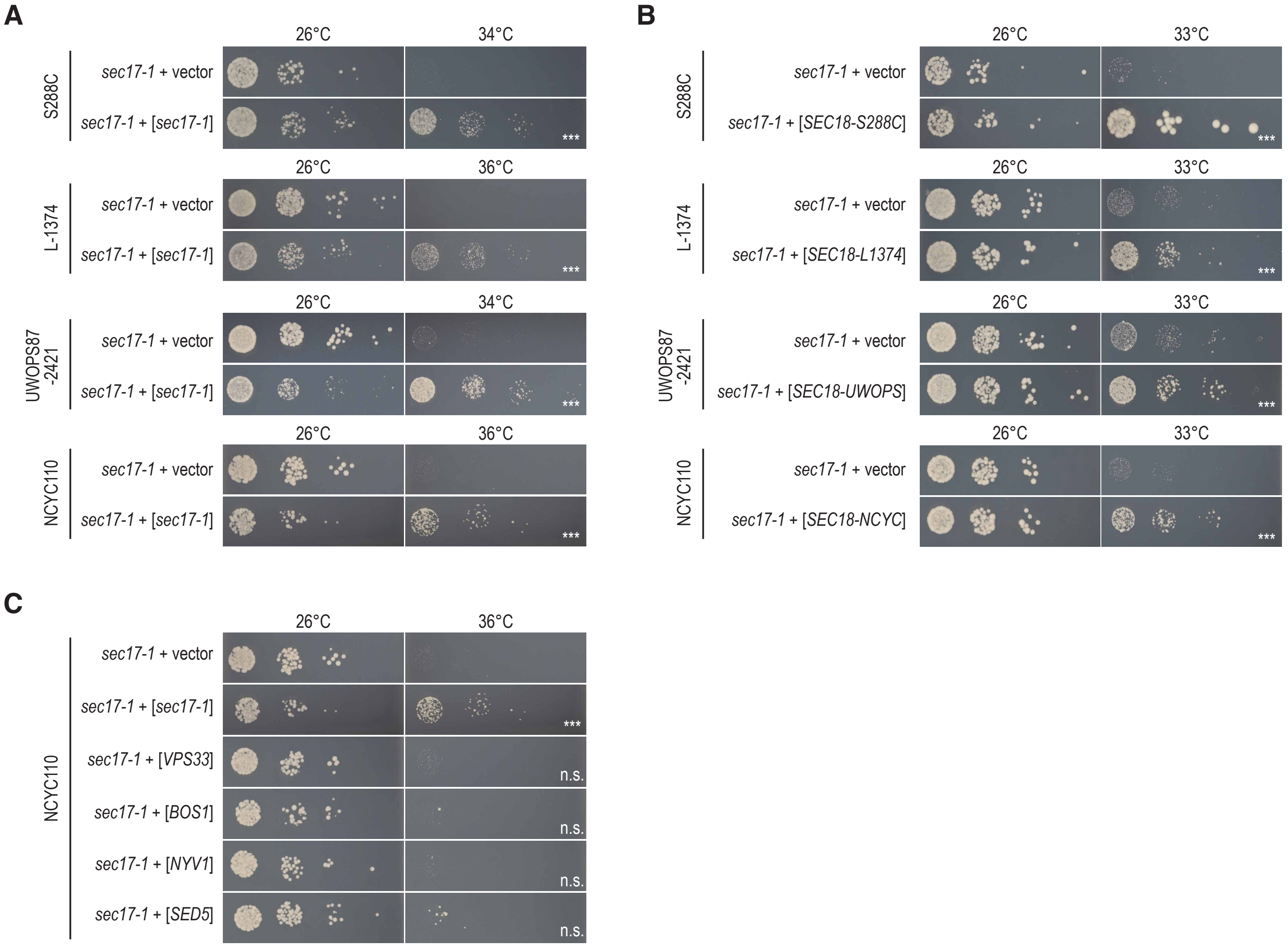

Fig. S2. Validation of *SEC17* suppressors. (**A-C**) Suppression of *sec17-1* TS strains by overexpression of genes located on aneuploid chromosomes: *sec17-1* (A) or *SEC18* (B), both located on chromosome II, or genes located on chromosome XII (C). Cultures of three independent transformants of the indicated strains were grown until saturation, and a series of ten-fold dilutions was spotted on SD –Ura plates. Plates were incubated at the indicated temperatures for two days. Plates were imaged, colony sizes were quantified, and statistical significance of size differences between suppressor candidates and corresponding controls was determined using Welch's t-tests. Pictures of one representative isolate are shown for each genotype. UWOPS = UWOPS87-2421; NCYC = NCYC110; * $p < 0.05$; ** $p < 0.005$; *** $p < 0.0005$; n.s., not significant.

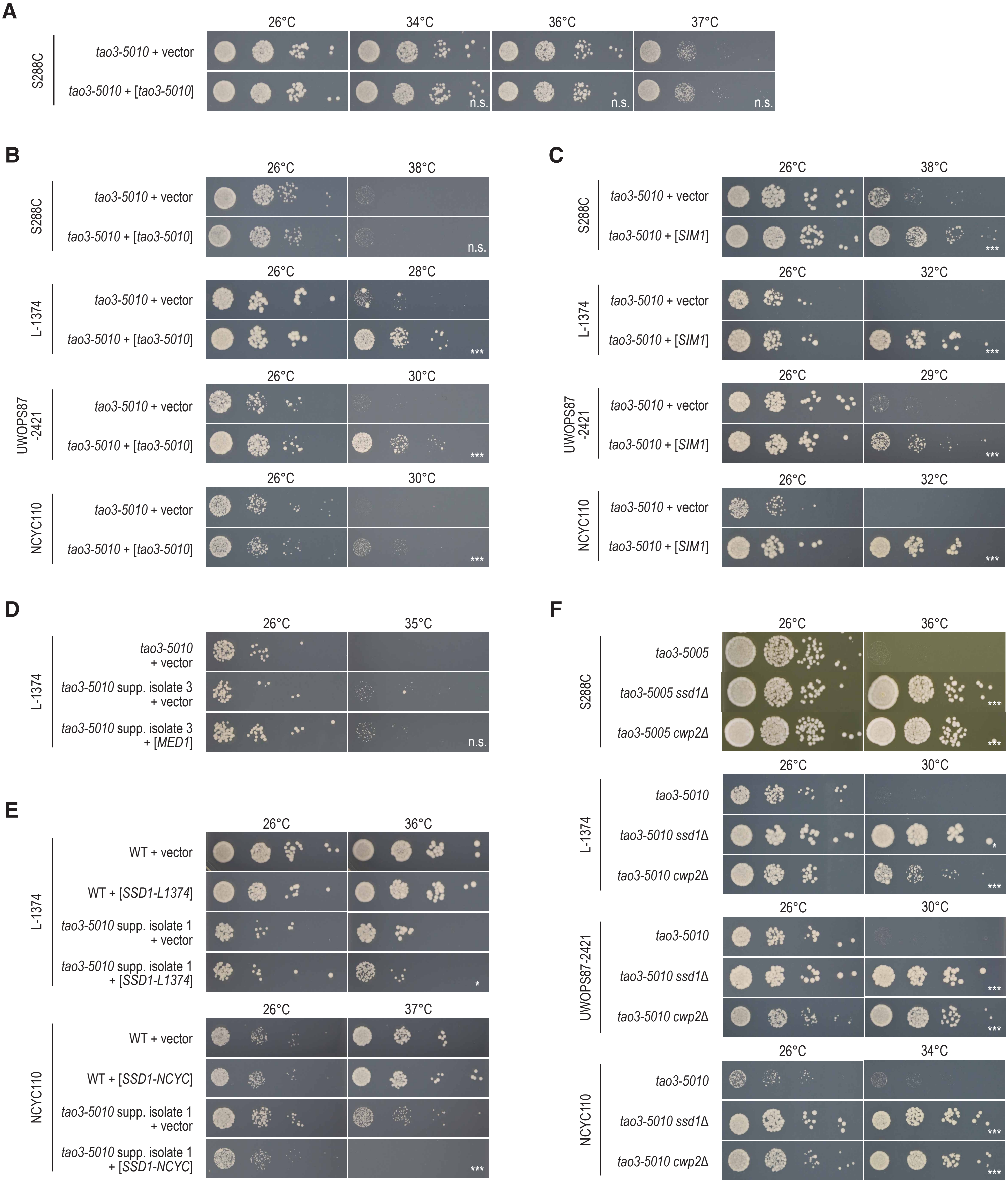

Fig. S3. Validation of *TAO3* suppressors. (**A-F**) Spot dilution assays of *tao3-5010* strains overexpressing *tao3-5010* (A, B), *SIM1* (C), *MED1* (D), or *SSD1* (E), or deleted for *SSD1* or *CWP2* (F). Cultures of two to three independent isolates of the indicated strains were grown until saturation, and a series of ten-fold dilutions was spotted on SD –Ura +NAT (A, B: S288C), SD –Leu (C), SD –Ura (B and F: L-1374, UWOPS87-2421, NCYC110; D; E), or YPD +NAT (F: S288C). Plates were incubated at the indicated temperatures for two days. Plates were imaged, colony sizes were quantified, and statistical significance of size differences between suppressor candidates and corresponding controls was determined using Welch's t-tests. Pictures of one representative isolate are shown for each genotype. * $p < 0.05$; ** $p < 0.005$; *** $p < 0.0005$; n.s., not significant.

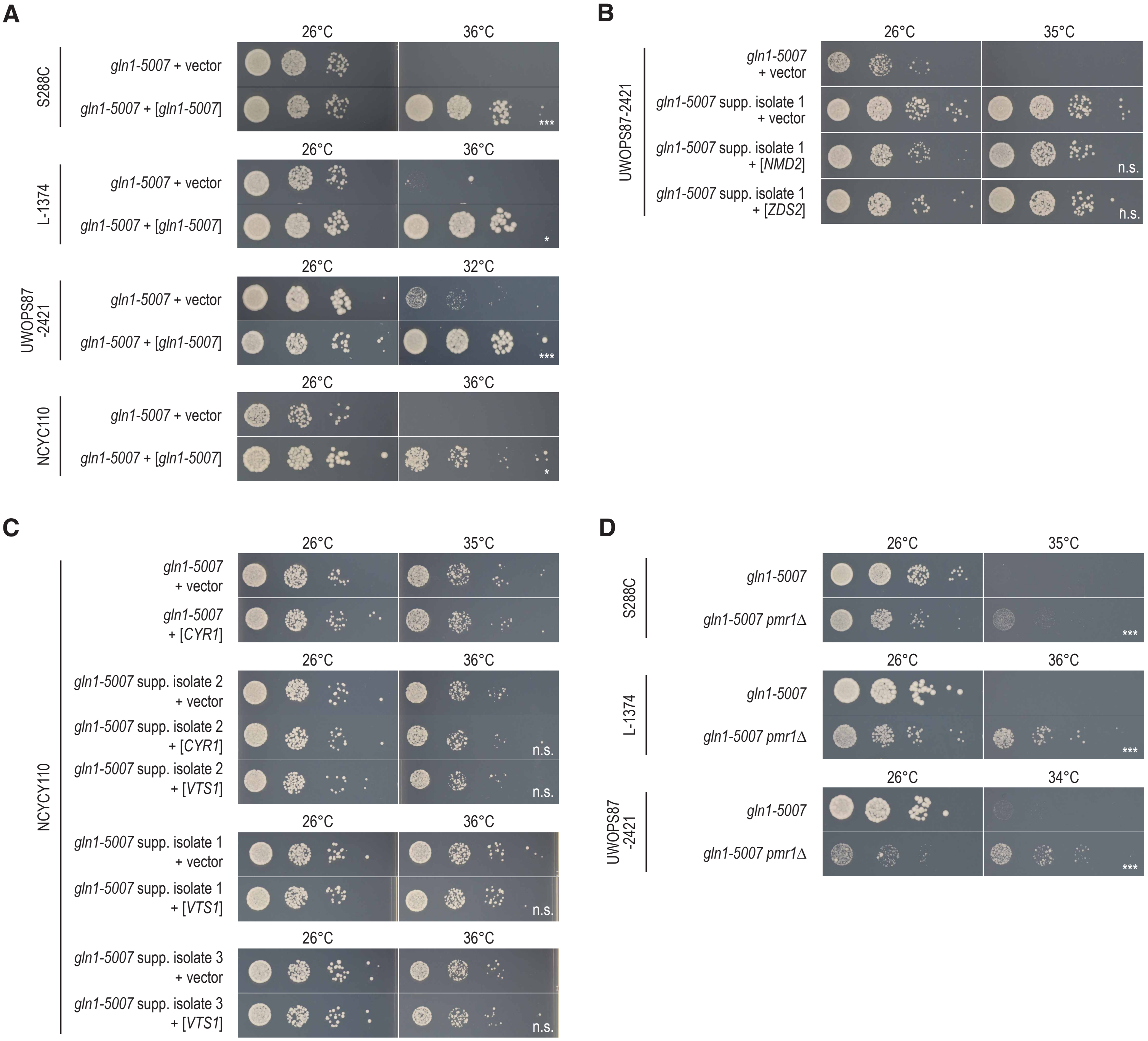

Fig. S4. Validation of *GLN1* suppressors. (**A**-**D**) Spot dilution assays of *gln1-5007* strains overexpressing *gln1-5007* (A), *NMD2* (B), *ZDS2* (B), *CYR1* (C), or *VTS1* (C), or deleted for *PMR1* (D). Cultures of two to three independent isolates of the indicated strains were grown until saturation, and a series of ten-fold dilutions was spotted on SD –Ura plates. Plates were incubated at the indicated temperatures for two days. Plates were imaged, colony sizes were quantified, and statistical significance of size differences between suppressor candidates and corresponding controls was determined using Welch's t-tests. Pictures of one representative isolate are shown for each genotype. * $p < 0.05$; ** $p < 0.005$; *** $p < 0.0005$; n.s., not significant.

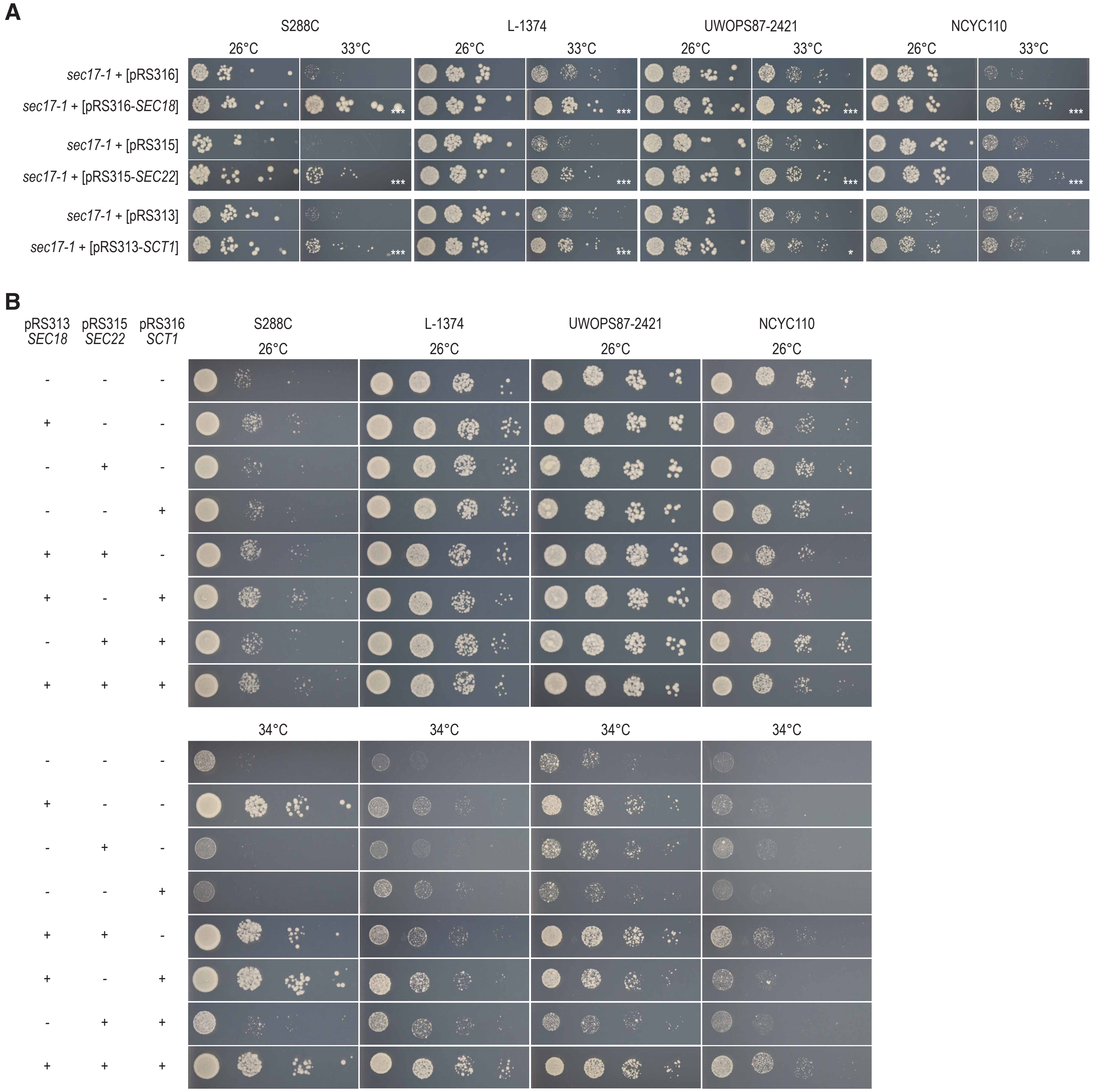

Fig. S5. Multiple genes can contribute to the suppression phenotype. (**A**) Validation of *sec17-1* suppressors using pRS-plasmids. *SEC18, SEC22*, or *SCT1* were cloned into pRS-plasmids and transformed into S288C, L-1374, UWOPS87-2421, and NCYC110 *sec17-1* strains. In each case, the *SEC18*, *SEC22*, and *SCT1* overexpression alleles matched the genetic background in which they were transformed, such that S288C was transformed with S288C alleles and L-1374 with L-1374 alleles, etc. Cultures of three independent transformants of the indicated strains were grown until saturation, and a series of ten-fold dilutions was spotted on SD –Ura (*SEC18* overexpression), SD –Leu (*SEC22* overexpression), or SD –His (*SCT1* overexpression). Plates were incubated at the indicated temperatures for three days. Plates were imaged, colony sizes were quantified, and statistical significance of size differences between suppressor candidates and corresponding controls was determined using Welch's t-tests. Pictures of one representative transformant are shown for each genotype. * $p < 0.05$; ** $p < 0.005$; *** $p < 0.0005$; n.s., not significant. (**B**) Spot dilution assays as in (A), but using combinations of *SEC18*, *SEC22*, and/or *SCT1* plasmids. Plates were incubated at the indicated temperatures for two days. + = strains were transformed with the indicated plasmids. - = strains were transformed with the corresponding empty vectors. Quantification and statistical analysis are shown in Figure 5.
